# Growth Parameters Impairment in Patients with Food Allergies

**DOI:** 10.1155/2014/980735

**Published:** 2014-05-15

**Authors:** Larissa Carvalho Costa, Erica Rodrigues Rezende, Gesmar Rodrigues Silva Segundo

**Affiliations:** Pediatrics Department, Federal University of Uberlândia, Campus Umuarama, Avenida Para 1720, 38400-920 Uberlândia, MG, Brazil

## Abstract

*Background and Aims.* Food allergy (FA) is a common disease that is rapidly increasing in prevalence for reasons that remain unknown. *Objective.* The aim of this study was to analyze the clinical characteristics and anthropometric data of patients with food allergies followed in a tertiary centre of allergy and immunology. *Methods.* A retrospective study was performed that assessed the data records of patients with food allergy diagnosis, covering a period from February 2009 to February 2012. *Results.* 354 patients were evaluated in the period; 228 (69.1%) patients had a confirmed FA diagnosis. The *z*-scores for weight-for-age, height-for-age, and body mass indices-for-age showed lower significant values in the FA group compared with the non-FA group by Mann-Whitney test, with significance values of *P* = 0.0005, *P* = 0.0030, and *P* = 0.0066, respectively. There were no statistical differences in sex, gestational age, birth type, breastfeeding period, and age of introduction of complementary formulas based on cow milk protein between groups. *Conclusion.* FA patients had a lower growth rate in comparison with patients without FA. The early recognition of food allergies with the establishment of protein-implicated diet exclusion, in association with an adequate nutrient replenishment, is important to reduce the nutritional impact of food allergies.

## 1. Introduction 

Food allergy (FA) is a common disease that is rapidly increasing in prevalence for reasons that remain unknown. Recent estimates suggest that around 17 million people in Europe suffer from allergies triggered by foods such as milk, eggs, peanuts, tree nuts, or seafood, and an increasing number are seeking treatment through primary care and hospital emergency departments [1]. A recent national survey of allergies in the United States showed an increase in the prevalence of food allergies from 3.4% in 1997–1999 to 5.1% in 2009–2011 [2].

FA can have a significant effect on an individual's quality of life and physical functioning and can also be costly in terms of medical visits and treatments [1]. Food allergies manifest various symptoms in the skin, gastrointestinal tract, and airways as a result of adverse responses to a food protein via IgE-mediated or non-IgE-mediated immune mechanisms. Allergic responses to food present as inflammation due to cellular responses activated against the food allergen [3].

There is a lack of information on the role of nutrition versus only food avoidance in the management and natural history of food allergy. Little information is also known about the effect of a nutrition consultation in this process. Furthermore, the role of the dietician and the diagnostic and therapeutic value of the elimination diet has not been established and extensively investigated [4].

The aim of the present study is analyze the clinical characteristics and nutritional status of patients with food allergies followed in a tertiary centre of allergy and immunology.

## 2. Methods 

This was a retrospective study that assessed the data records of patients who were evaluated in the food allergy out-patient clinic of the Clinical Hospital of Federal University of Uberlandia, between February 2009 and February 2012.

Data from age, sex, gestational age, delivery birth type, breastfeeding period, age of introduction of complementary formulas based on cow milk protein, anthropometric parameters, and foods implicated in allergies were obtained from the patient records.

The diagnosis routine of food allergy was performed based on the history and laboratory tests (specific IgE, skin prick test). Since there are no effective laboratory methods for the diagnosis of non-IgE-mediated FA, suspected in patients with symptoms suggestive of proctocolitis and enterocolitis, or in patients with diagnosis of eosinophilic esophagitis and atopic dermatitis moderate to severe, an elimination diet without a suspected allergenic protein and subsequent oral food challenge, which was open for children younger than 3 years and double-blind for children above 3 years, was used for diagnosis [5, 6].

The anthropometric data of patients seen for the first time was evaluated according to *z*-scores for weight-for-age, height-for-age, and body mass index (BMI). WHO-Antro software version 3.2.2, January 2011, available from (http://www.who.int/childgrowth/software/en/) was used to calculate *z*-scores and the World Health Organization growth charts were used as reference values [7].

The Kolmogorov-Smirnov test was used to determine whether variables were normally distributed. For continuous variables, groups were compared using the Mann-Whitney test. The Fisher exact test and Chi-square for trend were used for categorical variables. The level of significance for all statistical tests was 2-sided, *P* < 0.05. All analyses were conducted using Graph Pad Prism 5.0 (La Jolla, California, USA). The study was approved by the Human Research Ethics Committee of Federal University of Uberlandia.

## 3. Results 

In the period studied, the medical files showed that 389 different patients were evaluated in the out-patient clinic of food allergy. Of these, 35 patients did not undergo any consultation or medical records did not present data regarding the query. Among the 354 patients that were evaluated by physicians in our unit, 228 (69.1%) had a confirmed diagnosis of food allergy, while the diagnosis of food allergy was excluded in the remaining 126 (30.9%).

The median age of patients with an FA diagnosis was 10 months (1–193 months) while the group without FA was 36.5 months (1–216 months). There was a significant difference between the ages of the groups (*P* < 0.0001 by Mann-Whitney test). There were no statistical differences in sex, gestational age, birth type, breastfeeding period, and age of introduction of complementary formulas based on cow milk protein, as shown in Table 1.

The most frequent symptoms in patients with food allergy were nausea and vomiting in 118 (51.7%), abdominal pain in 104 (45.6%), diarrhea in 73 (32.0%), fresh rectal bleeding in 71 (31.1%), failure to thrive in 55 (24.1%), urticaria in 50 (21.9%), constipation in 19 (8.3%), dysphagia in 8 (3.5%), and food aversion in 8 (3.5%). The diagnosis of non-IgE-mediated allergy was performed in 168 (73.68%), while the diagnosis of IgE-mediated food allergy was performed in 75 (32.89%). The diagnosis of mixed IgE and non-IgE-mediated food allergy was done in 15 (6.57%). The foods implicated in food allergies are presented in Table 2.

The *z*-scores for weight-for-age, height-for-age, and body mass indices- (BMI-) for-age showed less significant values in the FA group compared with the non-FA group by Mann-Whitney test, with values of *P* = 0.0005, *P* = 0.0030, and *P* = 0.0066, respectively (Table 1).

Indeed, 18.4% presented with a low weight-for-age *z*-score (<−2.0 standard deviation (SD)), 15.9% with a low height-for-age *z*-score (<−2.0 SD), and 15.4% with a low BMI-for-age *z*-score (<−2.0 SD). A significant difference in low levels (<−2.0 SD) by Fisher exact test was found between the FA and non-FA groups only in height-for-age *z*-score (*P* = 0.0189), although the weight-for-age *z*-score analysis showed a *P* value that was close to significance (*P* = 0.0549). When we separated the groups with *z*-score less than −2 and up, there is no difference relative to the numbers of implicated food allergens. In BMI-for-age *z*-score less than −2, 16 (51.61%) of patients had one food allergy, 9 (29.03%) had two foods involved, and 6 (19.36%) had three or more foods, while in group with *z*-scores up −2, 117 (59.40%) had one food implicated, 36 (18.28%) had two foods, and 44 (22.32%) had three or more foods.

**Figure 1:**
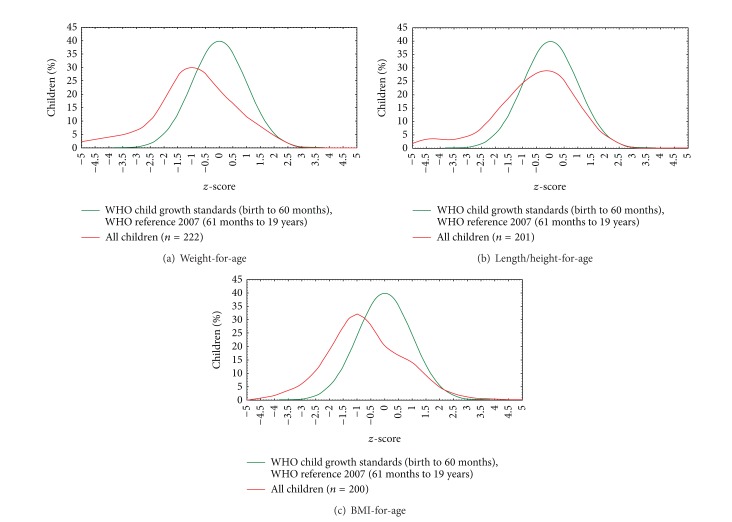
Comparison between *z*-scores reference curve from World Health Organization and patients with food allergy evaluated in the food allergy out-patient clinic from Clinical Hospital of Federal University of Uberlandia, Brazil. (a) Weight-for-age, (b) length/height-for-age, and (c) BMI- (body mass index-) for-age.

Figure 1 represents the comparison between *z*-scores of food allergy patients and a reference curve of growth in childhood. Food allergy patient curve is lower and presents a left variation in evaluation with reference to the three parameters (weight-for-age, height-for-age, and BMI-for-age) demonstrating the impairment in growth parameters.

## 4. Discussion 

The incidence and prevalence of food allergies are believed to be increasing in several countries. Dietary antigens induce a local hypersensitivity reaction impairing the intestine's barrier function, leading to the continuation of inflammation. The consequences of inflammatory responses may be severe and manifest as impaired growth, increased symptoms, and poor quality of life [4].

We did not find an association between sex, gestational age, delivery birth type, breastfeeding period, and age of introduction of complementary formulas based on cow milk protein with the development of FA. However, the number of medical records without any analyzed data was a limiting factor in data analysis. Approximately one-third of the patients did not have the studied data clearly outlined in their medical report, indicating that it is important to improve the completion of data in documents.

In the present study, cow's milk protein was responsible for FA in more than 90% of patients with non-IgE-mediated disease and more than 78% in patients with an IgE-mediated mechanism. Similarly to other studies, besides cow's milk, the foods most commonly implicated with allergy in infants were hen's eggs, soy, and wheat. In contrast to that found in other studies, allergy to corn protein was found in 13.59% of patients, demonstrating a peculiarity of this region where there is intense corn consumption due to cultural factors. Several patients had more than one food sensitization.

One study performed in Brazil and focused on infants with cow milk allergies showed that, among the 159 patients seen at first evaluation, 15.1% presented with a low weight-for-age *z*-score (<−2.0 SD), 8.7% with a low weight-for-height *z*-score (<−2.0 SD), and 23.9% with a low height-for-age *z*-score (<−2.0 SD) [8]. We also found similar data in the present study, as shown in the Results section. Another study conducted on 99 patients with food allergies showed that patients with a milk allergy or multiple food allergies are at greater risk of developing growth problems or inadequate nutrient intake.

In the present study, the control group was filled by patients that looked for the out-patient clinic with FA suspicion; then it is possible that patients could have another clinical disorder that affected their growth data and express less significant differences between FA and control group. Because of that, the comparative evaluation with WHO growth reference curves is important. The curves showed a clear impact in growth data found in FA group in all scores in comparison with reference values.

More than 25% of children in both groups consumed less than 67% of the dietary reference intakes for calcium, vitamin D, and vitamin E [9]. According to previous data, the present study showed lower *z*-scores in FA patients related to non-FA patients, suggesting difficulties in receiving adequate nutrient intake when a necessity to avoid specific proteins exists. This difficulty in establishing a diet and the presence of levels of *z*-score less than −2 apparently were related with absence of medical and nutritional information, once there are no significant correlation with number of the implicated foods in allergy.

We also believe that the presence of non-IgE-mediated FA promotes intestinal inflammation that can lead to difficulties in the absorption of other nutrients, which justifies the meeting of nutritional changes or even the admission of patients to hospital. These difficulties in a phase of life where there is a large increase in height and weight probably contributed to the difference in *z*-scores observed in patients with food allergies [10].

One limitation of this study in respect to nutritional status is the use only of height, weight, and BMI, but these are only data possible in medical charts. Another limitation of this study is the retrospective format. The cohort studies will effectively show more accurate data, but that does not cut the value of the present study.

In conclusion, FA patients had a lower growth rate in comparison with patients without FA. The early recognition of food allergies with the establishment of a diet excluding the implicated protein, in association with an adequate nutrient replenishment, is important to reduce the nutritional impact of food allergies.

## Figures and Tables

**Table 1:** Data from patients evaluated in food allergy out-patient clinic from Clinical Hospital of Federal University of Uberlandia, Brazil.

Data	Groups		*P* value
	Food allergy (*n*: 228)	Nonfood allergy (*n*: 126)	
Age (median in months)	10	36.5	<0.0001*
Sex—male (%)	120 (52.63)	75 (59.53)	0.5067**
Birth delivery—*n* (%)			
Vaginal	24 (10.53)	13 (10.32)	
Cesarean	128 (56.14)	59 (46.82)	0.1882***
ND	76 (33.33)	54 (42.86)	
Gestational age—*n* (%)			
Preterm	36 (15.79)	13 (10.32)	
Term	87 (38.16)	50 (39.68)	0.2235***
Postterm	1 (0.44)	0 (0.00)	
ND	104 (45.61)	63 (50.00)	
Breastfeeding—*n* (%)			
No breastfeed	37 (16.23)	9 (7.14)	
<1 month	54 (23.68)	25 (19.84)	
1–6 months	92 (40.35)	34 (26.98)	0.5363***
>6 months	3 (1.32)	2 (1.59)	
Only breastfeed	1 (0.44)	1 (0.80)	
ND	41 (17.98)	55 (43.65)	
Onset use of complementary formulas—*n* (%)			
<1 month	81 (35.53)	24 (19.04)	
1–6 months	73 (32.02)	23 (18.25)	
6–12 months	20 (8.77)	7 (5.56)	0.1154***
>12 months	2 (0.88)	0 (0.00)	
No use	8 (3.51)	7 (5.56)	
ND	44 (19.29)	65 (51.59)	
*z*-score weight × age			
Median (interval)	−0.95 (−5.30–2.25)	−0.30 (−4.77–4.80)	0.0005*
*z*-score height × age			
Median (interval)	−0.41 (−5.04–2.81)	−0.01 (−4.78–2.55)	0.0030*
*z*-score BMI × age			
Median (interval)	−0.85 (−6.20–3.59)	−0.33 (−3.06–6.55)	0.0053*

*Mann-Whitney test.

**Fisher's exact test.

***Chi-square test for trend.

BMI: body mass indices.

**Table 2:** Food implicate with Non-IgE- and IgE-mediated food allergens in patients evaluated in food allergy out-patient clinic from Clinical Hospital of Federal University of Uberlandia, Brazil.

Food implicate	Allergy type		Total (*n*: 228)
	Non-IgE-mediated (*n*: 168)	IgE-mediated (*n*: 75)	
Cow's milk	152 (90.48%)	59 (78.67%)	211 (92.54%)
Hen's egg	23 (13.69%)	38 (50.67%)	61 (26.75%)
Soy	29 (17.26%)	13 (17.33%)	42 (18.42%)
Wheat	19 (11.31%)	10 (13.33%)	29 (12.72%)
Corn	23 (13.69)	8 (10.67%)	31 (13.59%)
Chicken	4 (2.38%)	2 (2.67%)	6 (2.63%)
Beef	3 (1.78%)	2 (2.67%)	5 (2.19%)
Pork	2 (1.19%)	2 (2.67%)	4 (1.75%)
Fish	2 (1.19%)	—	2 (0.88%)
Other foods	11 (6.55%)	6 (8.00%)	17 (7.46%)
